# Representation of behaviour change interventions and their evaluation: Development of the Upper Level of the Behaviour Change Intervention Ontology

**DOI:** 10.12688/wellcomeopenres.15902.2

**Published:** 2021-01-06

**Authors:** Susan Michie, Robert West, Ailbhe N. Finnerty, Emma Norris, Alison J. Wright, Marta M. Marques, Marie Johnston, Michael P. Kelly, James Thomas, Janna Hastings

**Affiliations:** 1 Centre for Behaviour Change, University College London, London, UK; 2 Research Department of Epidemiology & Public Health, University College London, London, UK; 3 ADAPT SFI Research Centre, Trinity College Dublin, Dublin, Ireland; 4 Aberdeen Health Psychology Group, University of Aberdeen, Aberdeen, UK; 5 Primary Care Unit, Institute of Public Health, University of Cambridge, Cambridge, UK; 6 UCL Institute of Education, University College London, London, UK

**Keywords:** Behaviour, behaviour change, ontologies, interventions, evidence synthesis, evaluation studies

## Abstract

**Background: **Behaviour change interventions (BCI), their contexts and evaluation methods are heterogeneous, making it difficult to synthesise evidence and make recommendations for real-world policy and practice. Ontologies provide a means for addressing this. They represent knowledge formally as entities and relationships using a common language able to cross disciplinary boundaries and topic domains. This paper reports the development of the upper level of the Behaviour Change Intervention Ontology (BCIO), which provides a systematic way to characterise BCIs, their contexts and their evaluations.

**Methods: **Development took place in four steps. (1) Entities and relationships were identified by behavioural and social science experts, based on their knowledge of evidence and theory, and their practical experience of behaviour change interventions and evaluations. (2) The outputs of the first step were critically examined by a wider group of experts, including the study ontology expert and those experienced in annotating relevant literature using the initial ontology entities. The outputs of the second step were tested by (3) feedback from three external international experts in ontologies and (4) application of the prototype upper-level BCIO to annotating published reports; this informed the final development of the upper-level BCIO.

**Results: **The final upper-level BCIO specifies 42 entities, including the BCI scenario, elaborated across 21 entities and 7 relationship types, and the BCI evaluation study comprising 10 entities and 9 relationship types. BCI scenario entities include the behaviour change intervention (content and delivery), outcome behaviour, mechanism of action, and its context, which includes population and setting. These entities have corresponding entities relating to the planning and reporting of interventions and their evaluations.

**Conclusions: **The upper level of the BCIO provides a comprehensive and systematic framework for representing BCIs, their contexts and their evaluations.

## Introduction

Behaviour change interventions (BCIs), their contexts and their evaluations are heterogeneous both in their content and in how they are represented and reported. As a result, evidence of what works may be obscured as it is difficult to synthesise evidence and make recommendations for real-world policy and practice ( Elliott *et al.*, 2014). Ontologies provide a means for integrating knowledge across disparate data types and research paradigms and reducing ambiguity in reporting. They have been widely used in the biological and medical domains to enable integration. For example, the Gene Ontology ( Ashburner *et al.*, 2000) was created for the purpose of unifying annotations of gene function across model organism databases and has since grown to become essential to the modern practice of data-driven large-scale genomic science.

Ontologies represent knowledge in a given domain by defining the entities within the domain and the relationships between them and, by using a common language, are able to cross disciplinary boundaries and topic domains ( Arp *et al.*, 2015). At the heart of any ***ontology*** are a set of entities that are arranged into a hierarchy from the general to the specific, starting from the upper level which uses general terms enabling semantic ***interoperability*** with other ontologies, and continuing down to those that are specific to the domain (see glossary of italicised terms, Table 1). Entities may correspond to any sort of thing that exists, including objects, attributes and events. They are associated with unique and unambiguous identifiers, definitions, a primary label and one or more synonyms where applicable. They may be further inter-related by additional relations which can extend to complex logical expressions ( Arp *et al.*, 2015; Hastings, 2017).

**Table 1.** Glossary.

Term	Definition	Source
**Annotation**	Process of coding selected parts of documents or other resources to identify the presence of ontology entities.	Michie *et al*., 2017.
**Basic Formal Ontology** **(BFO)**	An upper level ontology consisting of continuants and occurrents developed to support integration, especially of data obtained through scientific research.	Arp *et al*., 2015.
**Continuant**	Entities within an ontology that continue to exist over time, for example, objects and spatial regions.	Arp *et al*., 2015.
**Entity**	Anything that exists, that can be a continuant or an occurrent as defined in the Basic Formal Ontology.	Arp *et al*., 2015.
**GitHub**	A web-based platform used as a repository for sharing code, allowing version control.	https://github.com/
**Interoperability**	Two systems are interoperable if data coming from each system can be used by the other system. Note: An ontology is interoperable with another ontology if it can be used together with or re-uses parts from the other ontology	http://www.obofoundry.org/principles/fp-010-collaboration. html
**Issue tracker**	An online log for problems identified by users accessing and using an ontology.	BCIO Issue Tracker: https://github.com/ HumanBehaviourChangeProject/ontologies/issues
**Minimum Information** **for Reporting an** **Ontology (MIRO)** **guidelines**	The Minimum Information Required for reporting Ontologies guidelines aiming to facilitate completeness and consistency in ontology documentation and reporting.	Matentzoglu *et al*., 2018.
**OBO Foundry**	The Open Biological and Biomedical Ontology (OBO) Foundry is a collective of ontology developers that are committed to collaboration and adherence to shared principles. The mission of the OBO Foundry is to develop a family of interoperable ontologies that are both logically well-formed and scientifically accurate.	Smith *et al*., 2007; www.obofoundry.org/
**OBO Foundry** **principles**	Good practice principles of ontology development and maintenance intended as normative for OBO Foundry ontologies. Ontologies submitted to OBO Foundry are evaluated against them.	http://www.obofoundry.org/principles/fp-000-summary.html
**Occurrent**	Entities within an ontology that extend over time, for example, processes.	Arp *et al*., 2015.
**Ontology**	A standardised framework providing a set of terms for the consistent annotation (or “tagging”) of data and information across disciplinary and research community boundaries.	Arp *et al*., 2015.
**Parent class**	A class within an ontology that is hierarchically related to one or more child (subsumed) classes such that all members of the child class are also members of the parent class and all properties of the parent class are also properties of the child class	Arp *et al*., 2015.
**Versioning**	Ontologies that have been released are expected to change over time as they are developed and refined, leading to a series of different files. Consumers of ontologies must be able to specify exactly which ontology files they used to encode their data or build their applications and be able to retrieve unaltered copies of those files in perpetuity. Versioning is one of the OBO Foundry principles.	http://www.obofoundry.org/principles/fp-004-versioning.html
**Web Ontology** **Language** **(OWL)**	A formal language for describing ontologies. It provides methods to model classes of “things”, how they relate to each other and the properties they have. OWL is designed to be interpreted by computer programs and is extensively used in the Semantic Web where rich knowledge about web documents and the relationships between them are represented using OWL syntax.	https://www.w3.org/TR/owl2-quick-reference/

This paper introduces an ontology that provides a systematic way of describing and linking together entities in the domain of behaviour change interventions: the Behaviour Change Intervention Ontology (BCIO). It reports the development and structure of the Behaviour Change Intervention Ontology’s upper level, that is, the domain-specific entities and their relationships which provide a high-level classification of the components of a behaviour change intervention and serve as a starting point for developing the lower-levels of the BCIO.

## Ontologies

Ontologies have been developed for many scientific domains, including chemistry, anatomy, disease and biomedical investigations; many are brought together as an interoperable collection in the context of the Open Biological and Biomedical Ontology (OBO) Foundry ( Smith *et al.*, 2007). The *OBO Foundry* promotes collaboration and interoperability across domains through advocating shared guidelines and best practices for ontology development, and the provision of a common framework. This common framework consists in part of a system of computational infrastructure, such as the use of the standard ontology language ***Web Ontology Language (OWL)*** and a set of standards for assigning identifiers and metadata. It also consists of a shared common understanding of the basic divisions of types of ***entities*** in the world. This common understanding is implemented as the ***Basic Formal Ontology (BFO)*** ( Arp *et al.*, 2015; Grenon *et al.*, 2004; Smith & Grenon, 2004). BFO is a domain neutral ‘top level’ or ‘formal’ ontology, beneath which other ontologies such as the BCIO can be developed. Aligning a domain ontology to a top level ontology is not strictly essential, but it supports the objectives of clarity and interoperability by basing developments on a shared foundation. While there are several different candidate top level ontologies to choose from (e.g. DOLCE ( Gangemi *et al.*, 2002), SUMO ( Pease *et al.*, 2002)), BFO is the one that has been adopted by the widest range of scientific ontologies and is recommended by the OBO Foundry ( Arp *et al.*, 2015; Smith *et al.*, 2007).

BFO recognises a fundamental distinction between universals and particulars, that is, between classes or generalities on the one hand and individual specific entities on the other. The subject matter in scientific ontologies, for the most part, is restricted to universals (classes of entity). BFO divides these universals or entities into two categories: ***continuants***, objects and spatial entities that continue to exist as the same individual entity over time, such as a population or clinical setting, and ***occurrents***, events or processes such as the implementation of a behaviour change intervention that occur or happen in time ( Arp *et al.*, 2015). This is a fundamental distinction that puts, for example, molecules on the one side and chemical reactions on the other; human beings on the one side and conversations on the other. Entities of both of these types form the subject matter of scientific investigations, and therefore both are needed for a rich description of the subject matter in any given domain.

In the hierarchy of continuants, the most important distinction is between those entities whose existence is not dependent on another entity, and those entities that require some other entity for their existence and continued manifestation. For example, a population is independent, while a population size needs to be borne by a population in order to exist and be manifested. Continuants that do not depend on any other entities are called “independent continuants”, while those that need another entity in order to exist, on which they depend, are called “dependent continuants”. Paradigmatic examples of independent continuants are objects -- connected, distinguishable unities such as a cell or a human being -- and object aggregates, or groups of objects, such as a population. For any independent continuant, there can be many dependent continuants that depend on it ( Arp *et al.*, 2015).

The ***Minimum Information for the Reporting of an Ontology (MIRO)*** guidelines ( Matentzoglu *et al.*, 2018) highlight the need for ontology developers to describe in detail aspects of ontology development such as motivation for development, scope and development community, methods of knowledge acquisition and managing change in the ontology. These guidelines motivate our discussion in the sections that follow.

## Development of the Behaviour Change Intervention Ontology (BCIO)

The protocol for the Human Behaviour-Change Project, for which the BCIO has been developed, can be found at https://doi.org/10.1186/s13012-017-0641-5 ( Michie *et al.*, 2017). The overall aim of the Human Behaviour-Change Project is to automate evidence searching, synthesis and interpretation to rapidly address questions from policy-makers, practitioners and others who want to know answers to questions that are variants of ‘ *What works, compared with what, how well, with what exposure, with what behaviours (for how long), for whom, in what settings and why?’*. To achieve this, evidence needs to be organised ontologically, i.e. associated with a shared formal description of entities and relationships capturing domain knowledge in order to enable aggregation and semantic querying.

This paper reports the development of the upper level of the BCIO, which characterises BCIs, their contexts and their evaluation. The aim is to create a stable, upper-level structure to populate the remainder of the BCIO in order to:

1. Help structure thinking and communication about BCIs;
2. Enable working across domains and disciplines by providing a common language to connect different epistemologies and terminologies (‘interoperability’);
3. Organise evidence to facilitate more sophisticated synthesis than is possible without an ontological approach, and inferences from synthesized evidence.

It is intended that the BCIO will be:

1. Extensive but recognise that it will not be comprehensive: for example, there may be aspects of context other than population and setting that independently influence the effects of interventions on behaviour;
2. Computer-readable to enable the application of Artificial Intelligence, including machine learning, to facilitate evidence synthesis and interpretation, and generation of new hypotheses and recommendations.

## Methods

**Figure 1.**
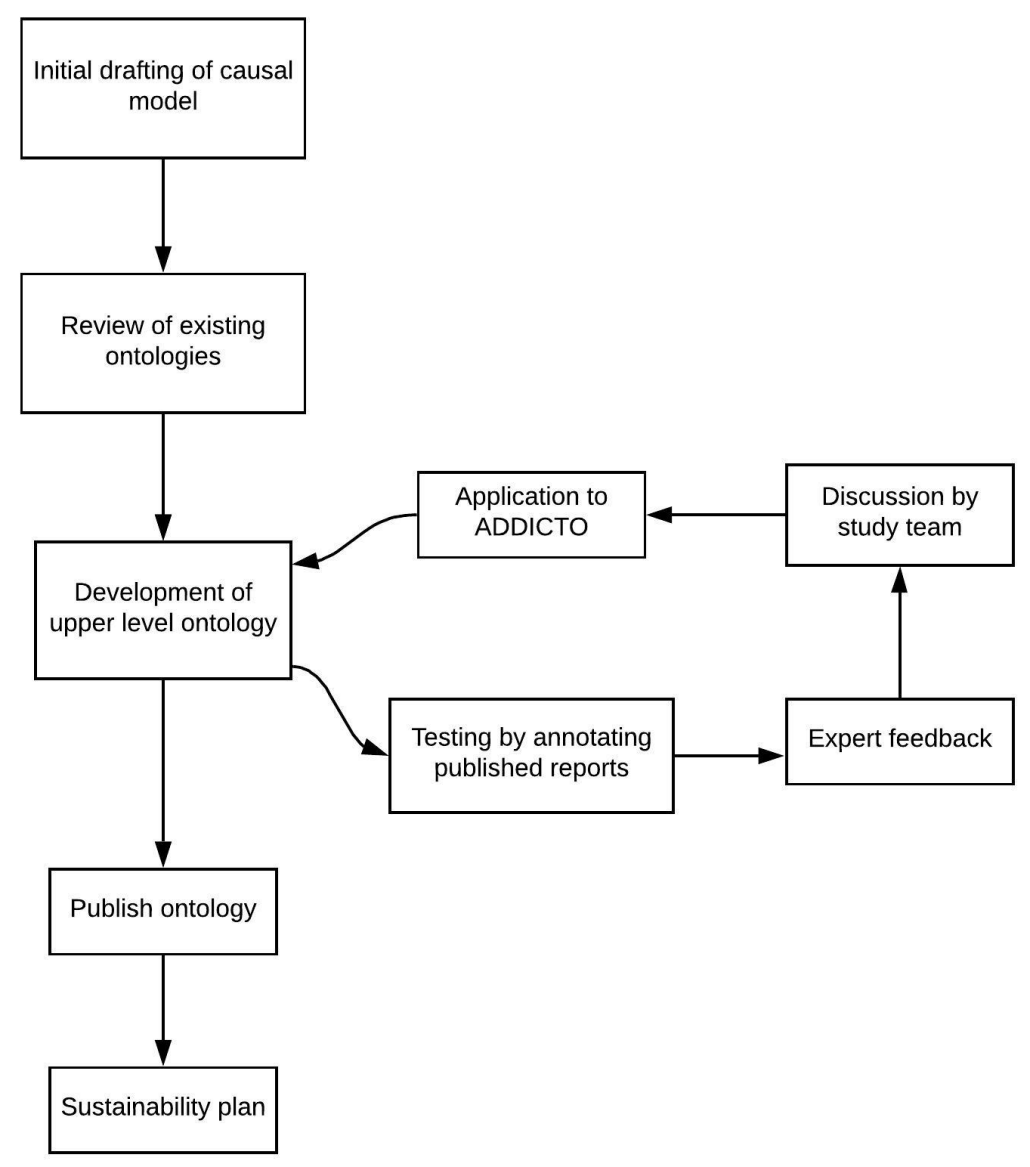
Stages of development of the upper-level Behaviour Change Intervention Ontology.

Development was undertaken in a number of steps, summarised in Figure 1 and described below.

## Initial drafting of entities and relationships in a causal model

This step established a causal model to predict how BCI outcomes are achieved in intervention evaluation studies. The scope of entities was considered in relation to the main research question of the project. ‘What intervention(s) work, compared with what, how well, with what exposure, with what behaviours, for how long, for whom, in what settings and why?’. Authors SM and RW discussed a basic structure of key entities and causal relationships, drawing on knowledge of theories and evidence about behaviour change and their experience of BCIs and evaluations. They also drew on three generic frameworks: Cochrane’s PICO ontology for systematic reviews (Population, Intervention, Comparison, Outcome), the Template for Intervention Description and Replication (TIDieR) ( Hoffmann *et al.*, 2014) and CONSORT guidelines for reporting clinical trials ( Schulz *et al.*, 2010). The basic structure was discussed with the wider research team of behavioural and social science experts. 

## Review of existing ontologies

A scoping review was conducted to establish whether an ontology of BCIs existed and whether existing similar ontologies contained entities related to human behaviour change that could be drawn into the upper-level BCIO (Full methods and results of this review published in Norris *et al.*, 2019). An extensive search via the Ontology Look-up Service and BioPortal was undertaken to identify entities related to behaviour change intervention evaluation studies that could be incorporated. Where possible, external content was incorporated using the Minimum Reporting Information to Reference an External Ontology (MIREOT) approach ( Courtot *et al.*, 2011). The causal model was converted into an ontology format, with entities linked to the BCI (the *BCI scenario*) differentiated from those linked to its evaluation (the *BCI evaluation study*).

## Data-driven development: Testing by annotating published reports

To test the applicability of the *BCI scenario* portion of the ontology to interventions described in reports and to check for overlap, missing entities and relationships at the upper level, interventions described in ~100 published reports of evaluations were *annotated*. These evaluation reports were randomly selected from a large dataset of published behaviour change intervention evaluation reports covering a range of behaviours, generated as part of wider research carried out at the Centre for Behaviour Change, University College London.

Reports were manually annotated independently by pairs of researchers. Entities or relationships between entities that could not be organised according to the existing structure of the upper level ontology but were considered potentially relevant were noted. The Human Behaviour-Change Project (HBCP) behavioural science team met regularly to discuss issues that arose from ***annotations*** and to resolve discrepancies in annotation. Differences between annotators in the way the ontology was used to annotate the reports were discussed and reconciled by the pairs of annotators. Uncertainties, new issues and challenges in applying the ontology were documented and discussed with the full HBCP team, including the ontology consultant. The methods used to develop the lower-level ontologies are available as *Extended data* at https://osf.io/dz8hu/ ( West *et al.*, 2020) and in the ontology methods paper accompanying this collection in *Wellcome Open Research* ( Wright *et al.*, 2020).

Reports in another domain, addiction, were also examined, taken from a database of reports used in developing an Addiction Ontology (AddictO) that is being developed in parallel with the BCIO. AddictO is an ontology for all aspects of addiction and its treatment that is being developed under the auspices of the Society for the Study of Addiction. More than 250 abstracts published in the previous two years in the two main generalist addiction journals, and selected in date order, were annotated to extract entities, 53% of which were determined to be within scope for the BCIO as they related to interventions and their evaluations. The process of extracting entities from addiction abstracts and ensuring that they could be adequately represented informed the development of the upper-level BCIO. 

## Expert feedback

The initial draft of the upper level of the BCIO was critically examined by six senior members of the HBCP behavioural science team (with backgrounds in psychology and sociology) and the study ontology expert. When the ontology had reached a sufficiently stable point in its development this was followed by feedback from three external international experts in ontologies. Experts were individuals with extensive experience and publication records in ontology development. Four experts were approached via email to participate, but one expert was unable to take part due to other commitments.

These three experts were asked to provide feedback on whether: 1) the entity names were clear; 2) the definitions were non-overlapping and without redundancy; 3) the relationships between the entities were suitable, such as being aligned with the types of relationships used in other upper-level ontologies; and 4) if the overall structure was clear. To assess whether they agreed with the statements, the experts were asked to respond with “Yes”, “To Some Extent” or “No”. They were also requested to provide justification for each of their responses. They were given the opportunity to provide additional comments on any aspect of the upper-level ontology. The expert feedback was used to refine both the upper and lower levels of the ontology.

## Discussion by study team

The expert feedback was also discussed by the research team to make the suggested changes by the experts where deemed appropriate. The team drew on BFO terminology to define entities and their relationships as a way of testing the upper-level BCIO and adjusted where necessary. Changes that were straightforward to implement were made. Comments that were more complex were discussed with the project ontology expert consultant. Definitions were amended following principles of good ontological definitions ( Michie *et al.*, 2019; Seppälä *et al.*, 2017). Experts’ comments along with the changes made and rationale for not incorporating are available as *Extended data* and at https://osf.io/h4sdy/ ( West *et al.*, 2020).

## Testing re-use in a separate ontology (AddictO)

As an ontology describing the domain of BCIs, a further test of the BCIO is to establish that it is applicable outside of its immediate development context. To this end, parts of the BCIO were adopted into AddictO. AddictO is in the preliminary stages of development but there are clear overlaps with the content in the BCIO insofar as that content relates to interventions and their evaluations, populations and settings. Behaviour change is one category of interventions used for the treatment of addiction, while other categories of treatment include pharmacological ones. Applying the BCIO to re-use in AddictO constituted a test of the definitions and interrelationships defined in the BCIO as to whether they were generally applicable and re-usable. Re-use of the BCIO in an external ontology helped to clarify which aspects of the BCIO were specific to behaviour change and which constituted a generic model for interventions and research within the social and behavioural sciences more broadly. 

## Creation of a sustainability plan

Ontologies are not static once created, but instead should be updated to reflect changes in the scientific consensus and suggestions from the wider scientific community ( http://www.obofoundry.org/principles/fp-016-maintenance.html). Therefore, a change management and version tracking strategy was developed in line with *OBO Foundry principles* of good practice ( http://www.obofoundry.org/principles/fp-004-versioning.html). Furthermore, in line with the OBO Foundry principle that ontologies should be made available in a common format, a computable version of the upper-level BCIO has been created using the OWL web ontology language. Making the BCIO available in this manner will facilitate further re-use, wider dissemination and interoperability with other ontologies.

## Results

The upper level BCIO entity labels, definitions and relationships to their ***parent classes*** are illustrated in Table 3. To bring the entities to life, the table also shows how the BCIO would apply to a specific BCI, the *Text2Quit* smoking cessation intervention, and its evaluation ( Abroms *et al.*, 2014). The results of each development step in the evolution of the ontology towards the final version shown in Table 3 are discussed further in the sub-sections that follow.

## Initial drafting of a causal model

**Figure 2.**
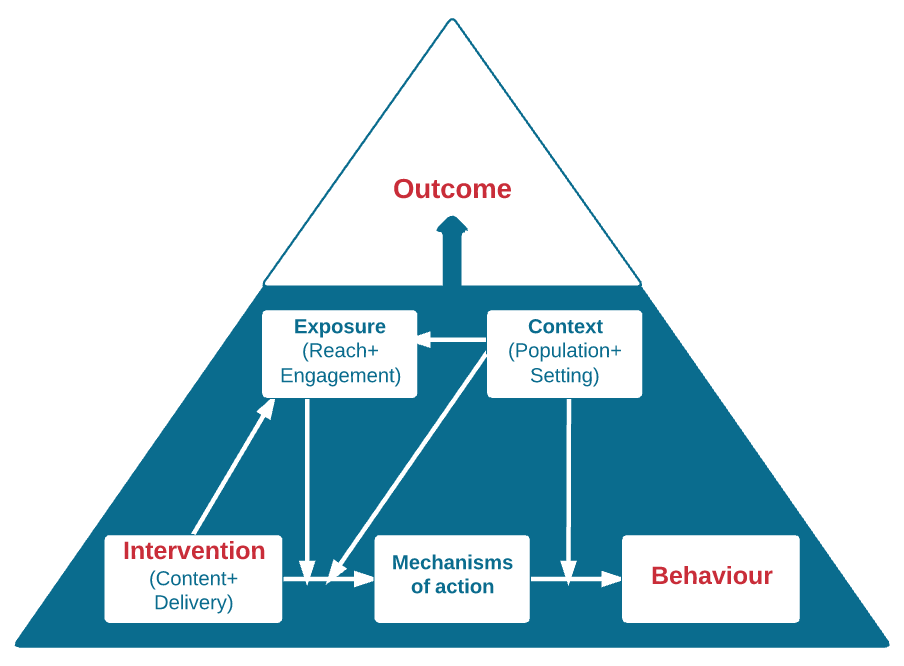
Initial schematic of upper-level Behaviour Change Intervention Ontology: scenario entities and causal connections.

The initial upper-level BCIO comprised a *BCI scenario* of 12 entities linked by arrows specifying the direction of the relationship without any specified ontological relationships: *Intervention*, *Content*, *Delivery*, *Mechanisms of action*, *Exposure*, *Reach*, *Engagement*, *Context*, *Population*, *Setting*, *Behaviour* and *Outcome* ( Figure 2).

## Review of existing ontologies

No entities from existing ontologies were selected for inclusion in the upper-level BCIO. However, the review identified several entities from existing ontologies that were used to populate the lower levels of the BCIO (see examples within our paper collection in the Intervention Setting Ontology & Population Ontology ( Norris *et al.*, 2020b). Moreover, terms from existing ontologies are used as parent terms providing the foundational classification structure for the upper-level BCIO. 

## Data-driven development: Testing by annotating study reports

An iterative process of annotating published study reports and team discussions resulted in identifying three delivery entities— *Source*, *Mode* and *Schedule*—as distinguishable processes within delivery, and a content entity alongside the description of the intervention type: *Dose*. This part of the process also gave rise to the concept of an intervention plan, such that *Fidelity* is the difference between planned and actualised intervention delivery and *Adherence* is the difference between planned and actualised engagement with the intervention by those targeted by the intervention. *Reach* is the difference between the BCI study sample and the planned BCI population.

## Expert feedback

Three external international ontology experts provided feedback on the first version of the upper-level ontology. They responded “Yes”, “No” and “To Some Extent” in responses to four questions, as shown in Table 2. They were asked to provide justifications for their responses, which are summarised below. The full feedback report is available as *Extended data* at https://osf.io/yj235/ ( West *et al.*, 2020).

**Table 2.** Experts’ responses to specific questions asked about the ontology.

	Questions for the Experts	Yes	To some extent	No
**1.**	The entity names were clear	-	2	1
**2.**	The definitions were non- overlapping and without redundancy	1	1	1
**3.**	The relationships were suitable	-	2	1
**4.**	The overall structure was clear	1	1	1

**Table 3.** BCIO entity labels, definitions and relationships to parent class.

Label (synonym)	Definition	Parent class	Elaboration	Example annotation for the Text2Quit intervention and its evaluation
**Behaviour change** **intervention scenario plan (BCI scenario** **plan)**	A plan that is realized in a BCI scenario process.	Plan (OBI)		The Text2Quit intervention team’s plan for running the intervention
**Behaviour change** **intervention scenario (BCI** **scenario)**	A process in which a BCI is applied in a given context, including BCI engagement and outcome behaviour.	Planned process (OBI)		Providing the Text2Quit intervention to smokers, including the extent to which smokers engaged with the intervention and the outcome behaviour of abstaining from smoking
**Intervention**	A planned process that has the aim of influencing an outcome.	Planned process (OBI)	Examples of interventions are putting health warnings on cigarette packets, providing free stop smoking services and banning smoking in public places.	The Text2Quit intervention
**Behaviour change** **intervention** **(BCI)**	An intervention that has the aim of influencing human behaviour.	Intervention	Involves use of products, services, activities, rules or environmental objects.	The Text2Quit intervention
**Behaviour change** **intervention content (BCI** **content)**	A planned process that is part of a BCI and is intended to be causally active in influencing the outcome behaviour.	Planned process (OBI) ^1^	Consists of BCTs that can be classified using a BCT taxonomy.	Behaviour Change Techniques in the Text2Quit intervention include problem solving, self-monitoring of behaviour, self-monitoring of outcomes of behaviour, reducing prompts and cues, distraction and pharmacological support
**Behaviour change** **technique (BCT)**	A planned process that is the smallest part of BCI content that is observable, replicable and on its own has the potential to bring about behaviour change.	Planned process (OBI)		Examples included self-monitoring of behaviour, problem solving and pharmacological support
**Behaviour change** **intervention dose (BCI** **dose)**	An attribute of BCI content that is its amount or intensity.	Process attribute	This is a disjunctive class that is not currently fully defined because specific BCI content instances may vary in intensity and amount in different ways.	The number of behaviour change techniques provided in each SMS message, the frequency and rate at which specific behaviour change techniques were repeated during the intervention.
**Process attribute**	An attribute of a process	Process profile		N/A
**Behaviour change** **intervention delivery (BCI** **delivery)**	A part of a BCI that is the process by which BCI content is provided.	Planned process (OBI)		The process by which the Text2Quit content is provided to participating smokers
**Behaviour change** **intervention mode of** **delivery (BCI mode of** **delivery)**	An attribute of a BCI delivery that is the physical or informational medium through which a BCI is provided.	Process attribute		The Text2Quit BCI is delivered through text messages, website and emails
**Behaviour change** **intervention schedule of** **delivery (BCI schedule of** **delivery)**	An attribute of a BCI that involves its temporal organisation.	Process attribute	Includes the start and end of the BCI and its parts.	Overall duration = 6 months. Participants received ﬁve SMSs on their quit date, c.2 SMSs/day in the week after the quit date and c.3 SMSs per week for the next 2 months, then <1 SMS per week
**Behaviour change** **intervention style of** **delivery (BCI style of** **delivery)**	An attribute of a BCI delivery that encompasses the characteristics of how a BCI is communicated.	Process attribute	An example is cold and distant vs. warm and accepting.	Not specified
**Behaviour change** **intervention tailoring (BCI** **tailoring)**	An attribute of a BCI that relates to selection or modification of the BCI according to attributes of members of the BCI population or BCI context.	Process attribute	It includes static tailoring that is based on characteristics of a member of a BCI population or BCI context at a single point in time and dynamic tailoring that can change as a function of characteristics assessed at multiple time points.	Messages were tailored to ﬁrst name, quit date, top three reasons for quitting, money saved by quitting, and use of quit-smoking medications. If participants reported smoking, they were routed into a relapse message protocol to support setting a new quit date
**Behaviour change** **intervention source (BCI** **source)**	A role played by a person, population or organisation that provides a BCI.	Role	This includes individual people, groups of people, and organisations.	Not specified
**Intervention outcome**	A process that is influenced by an intervention.	Process	Includes individual human behaviour, mental activity and physiological activity. Includes undesirable outcomes, such as treatment side effects, and unintended negative consequences of the intervention.	Not smoking for at least 30 days; not smoking for the last 7 days
**Outcome behaviour**	Human behaviour that is an intervention outcome.	Human behaviour		Abstaining from smoking for at least the last 30 days
**Behaviour change** **intervention context (BCI** **context)**	An aggregate of entities that are not dependent on the intervention but may influence the effect of a BCI on its outcome behaviour.	Object aggregate	Includes as part BCI population and BCI setting. Use of the word ‘may’ conveys a non- zero probability given available information.	The USA and its population in 2011 – 2013.
**Behaviour change** **intervention setting (BCI** **setting)**	An aggregate of entities that form the environment in which a BCI is provided.	Object aggregate	Includes as parts social setting and physical setting.	The overall environment in which the Text2Quit intervention was provided
**Behaviour change** **intervention social setting** **(BCI social setting)**	An aggregate of people with whom a BCI population interacts.	Human population		The population of the USA
**Behaviour change** **intervention physical** **setting (BCI physical** **setting)**	A physical environment in which a BCI is delivered.	Environmental system (ENVO)		Geographical location: USA (NB because the intervention was delivered via text messages to participants’ mobile phones, there was no specific intervention site)
**Human population**	An aggregate of people	Object aggregate		Adults living in the US who smoke cigarettes
**Behaviour change** **intervention population** **(BCI population)**	An aggregate of people who are exposed to a BCI.	Human population		Smokers, average age 35.7 years, 65.6% female, 78.1% with at least some college/ trade school education. Smoked an average of 17 cigarettes a day at the start of the study and had moderate levels of nicotine dependence.
**Behaviour change** **intervention mechanism** **of action (BCI mechanism** **of action)**	An attribute of the process by which a behaviour change technique influences the behaviour.	Process		Not specified in the Abroms *et al.* (2014) report. Examples could include processes such as increasing smokers perceived confidence they could quit or increasing the perceived benefits of quitting
**Behaviour change** **intervention engagement** **(BCI engagement)**	Individual human activity that enables a BCI to influence the outcome behaviour.	Individual human activity	Includes mental activities and behaviours.	Participants sending text messages back to the computerized messaging system
**Intervention evaluation** **study**	A research study that aims to assess attributes of an intervention with regards to their positive or negative value.	Research study (SEPIO)		Any study that aimed to assess the attributes of the Text2Quit intervention with regards to their positive or negative value
**Behaviour change** **intervention evaluation** **study (BCI evaluation** **study)**	An intervention evaluation study of a BCI scenario.	Intervention evaluation study		Any study evaluating the Text2Quit intervention in relation to the outcome behaviour, stopping smoking.
**Behaviour change** **intervention comparison** **evaluation study (BCI** **comparison evaluation** **study)**	A BCI evaluation study that involves comparison between two or more BCI scenarios to produce one or more BCI effect estimates.	Behaviour change intervention evaluation study	Comparison involves identifying differences between the entities in the scenarios.	Randomised controlled trial comparing the outcomes of the Text2Quit intervention to outcomes of providing self-help materials to produce an estimate of the Text2Quit intervention’s effect on stopping smoking
**Behaviour change** **intervention evaluation** **study plan (BCI evaluation** **study plan)**	A plan for a BCI evaluation study.	Plan (OBI)		The lead investigator’s plan for how the Text2Quit intervention would be evaluated
**Evaluation finding**	A data item that is the output of an intervention evaluation study.	Data item (IAO)		11.1% of smokers receiving the Text2Quit intervention abstained from smoking
**Behaviour change** **intervention evaluation** **finding (BCI evaluation** **finding)**	An evaluation finding that is the output of a BCI evaluation study.	Evaluation finding		The risk of quitting successfully was 2.22 for the Text2Quit group relative to the self-help control group
**Behaviour change** **intervention outcome** **estimate (BCI outcome** **estimate)**	A BCI evaluation finding that is about an outcome behaviour.	Behaviour change intervention evaluation finding	This includes as subclasses 1) type of outcome estimate (e.g. mean, percentage), 2) value of outcome estimate (e.g. 1.5 cigs per day, 23%), 3) uncertainty estimate type (e.g. 95% CI), and 4) uncertainty estimate value (e.g. 12.0%-45.0%).	1) Percentage abstinence in the Text2Quit group 2) 11.1% 3) the type of uncertainty estimate calculated around the value of 11.1% 4) the value of the uncertainty estimate around the value of 11.1% [NB an uncertainty estimate for the outcome estimate is not reported in the paper]
**Behaviour change** **intervention effect** **estimate (BCI effect** **estimate)**	A BCI evaluation finding that characterises the difference between BCI outcome estimates of two BCI scenarios.	BCI evaluation finding	This includes the following subclasses: 1) BCI effect estimate type -the type of statistic used to represent the difference (e.g. odds ratio, mean difference), 2) BCI effect estimate value – the datum that represents the difference (e.g. 1.35), 3) BCI effect estimate uncertainty type – the type of statistic used to represent the range of uncertainty of the value (e.g. 95% confidence interval see STATO), and 4) the BCI effect estimate uncertainty value - the datum representing the uncertainty (e.g. 1.20-1.55).	1) Relative risk 2) 2.2 3) 95% confidence interval 4) (1.16 – 4.26)
**Behaviour change** **intervention study** **investigator (BCI study** **investigator)**	A role played by a person that contributes substantively to production or reporting of a BCI evaluation study.	Role	What counts as substantively is subject to judgement. The level and nature of the contribution can be defined using the CReDiT taxonomy ( https://casrai.org/credit/).	Abroms and colleagues
**Behaviour change** **intervention study sample** **(BCI study sample)**	A population whose behaviour is studied in a BCI evaluation study.	Human population		The 503 smokers who met eligibility criteria, were randomized to receive either Text2Quit or the self-help intervention and responded to mobile phone number verification
**Behaviour change** **intervention scenario** **report (BCI scenario** **report)**	A report that describes a BCI scenario.	Report (IAO)		N/A
**Behaviour change** **intervention evaluation** **report (BCI evaluation** **report)**	A report that is a description of a BCI evaluation study.	Report (IAO)	Includes entities that stand in direct relation to the study e.g. authors, findings, funding, aims.	The report published as Abroms, L. C., Boal, A. L., Simmens, S. J., Mendel, J. A., & Windsor, R. A. (2014). A randomized trial of Text2Quit: a text messaging program for smoking cessation. *American Journal of Preventive* *Medicine*, *47*(3), 242-250.
**Behaviour change** **intervention evaluation** **study risk of bias or error** **(BCI study risk of bias or** **error)**	An information content entity that is about the likelihood of the BCI evaluation finding misrepresenting the outcome behaviour.	Information content entity (IAO)		Not reported in the Abroms *et al.* paper. In a systematic review ( Whittaker *et al.*, 2019) that included this study, its risk of bias was reportedly judged as low in all domains
**Individual human** **activity**	A process that is produced by a person.	Process		Using tobacco products; reading a text message
**Individual human** **behaviour**	Individual human activity that involves co-ordinated contraction of striated muscles controlled by the brain.	Individual human activity		Smoking cigarettes
**Population behaviour**	An aggregate of individual human behaviours of members of a population.	Process		The proportion of members of a population who haven’t smoked for at least the last 30 days.
**Human behaviour**	Individual human behaviour or population behaviour	Process	Also referred to in definitions as human behaviour or just behaviour.	Smoking cigarettes

[1] ENVO: Environment Ontology; IAO: Information Artifact Ontology; OBI: Ontology for Biomedical Investigations; SEPIO: Scientific Evidence and Provenance Information Ontology

*Clear entity names.* The two experts who agreed that the names were clear ‘to some extent’ noted that the clarity could be improved by avoiding using the acronym BCI in the entity names as the acronym “is only clear in the Behaviour Change Ontology” as there are other popular BCI acronyms such as “Brain-Computer Interface”. They also noted that some of the concepts seemed vague or unnecessary, such as, having both BCI comparison and BCI evaluation when just one term could be used. The expert who thought that the entity names lacked clarity stated that it was a mistake "to define a general term like *Population* as having a very narrow meaning" as it would reduce the ability in the future "to compare populations who had and who had not been part of a behaviour intervention context".

*Definitions non-overlapping and without redundancy.* “Circularity” for some definitions was noted, such as for population, context and engagement. The description of some terms (e.g. “outcome behaviour”) as a “Process” was questioned as “the description does not really justify this decision”.

*Suitable relationships.* Suggestions made by the experts were to adhere to specific rules of using ontological relationships such as a suggestion to follow “the all-some rule, so if A has-part B then all instances of A have some instance of B has part” to ensure that the most suitable definitions were selected for the entities. The experts were not clear on “why there is so much emphasis on part-whole relationships” and that there was no need “to introduce new object properties” but to instead re-use existing relations from other ontologies, e.g. the Relations Ontology (RO) ( Smith *et al.*, 2005)

*Clear overall structure.* Experts noted that due to the use of an external upper-level ontology (i.e., BFO) “the structure is mostly clear”, but that some of the “descendants of process, are difficult to intuitively associate with processes” due to the naming convention. It was also noted that the version of the ontology did “not seem to have enough depth” for the tasks of reasoning and making inference from the evidence it was organising.

## Discussion by study team

### BCIO

Team discussions highlighted the need for new entities which had not been considered previously, identified connections across entities when lower level terms were found to be repeated across multiple ontologies and informed changes to definitions when new additions to the lower levels meant that upper-level definitions no longer covered what was needed. The main changes that were discussed from the expert feedback concerned entity definitions. When the development team was satisfied with the entity definitions and relationships, the intervention part of the BCIO was shared among the wider project team, including the systems architects and computer scientists, for final discussion ( https://github.com/HumanBehaviourChangeProject/ontologies; Norris *et al.*, 2020a).

**Figure 3.**
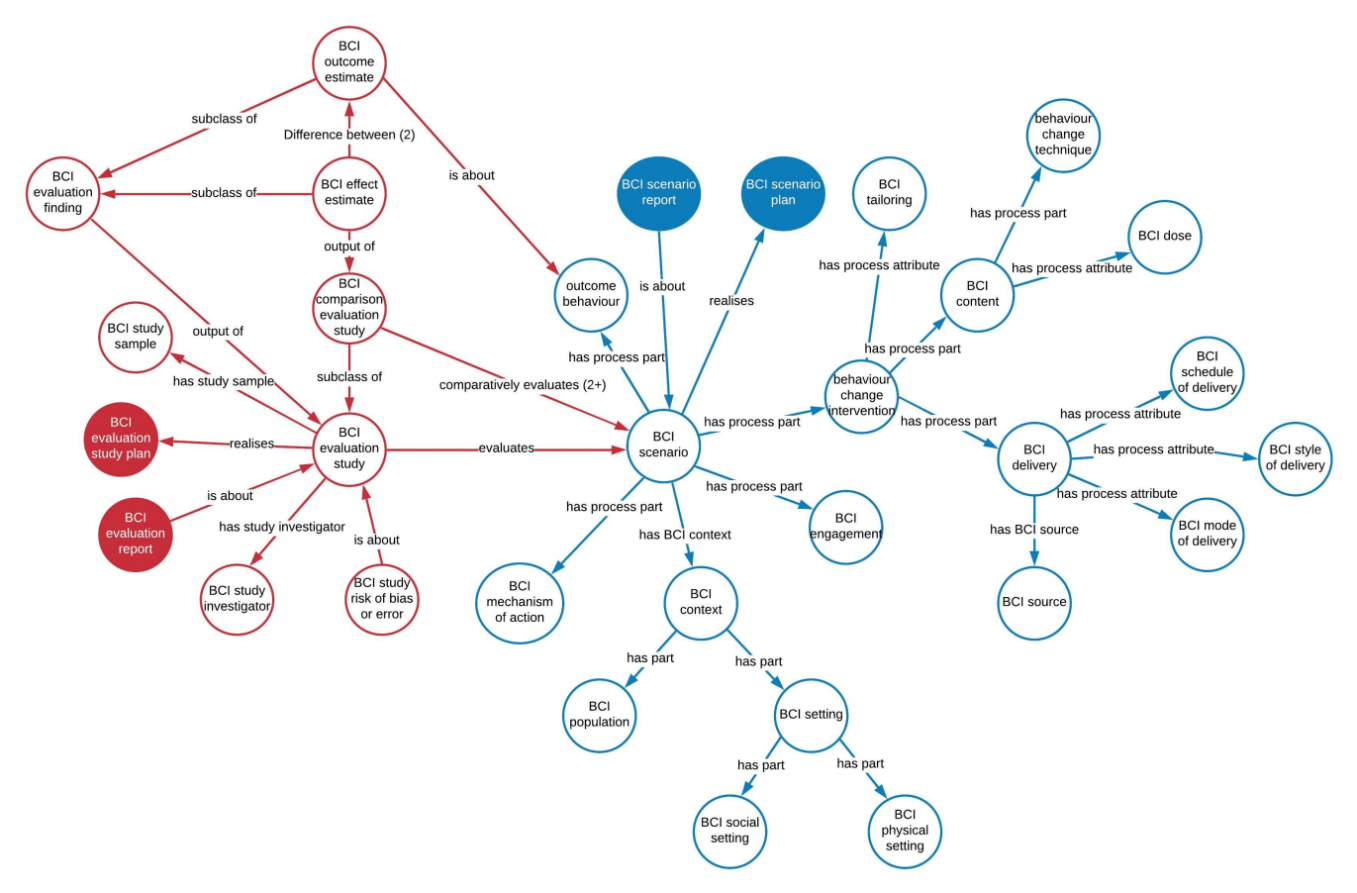
Entities and relationships that are central to the Behaviour Change Intervention Ontology (BCIO) V1.4.

The changes that were made following expert feedback and discussions by the study team can be identified by comparing the first conceptual version of the ontology ( Figure 2) and the final version of the BCIO ( Figure 3; Table 3). The resulting BCIO is divided into two parts 1) *BCI scenario* and 2) *BCI comparison evaluation study*. The *BCI scenario* has 21 entities: *BCI scenario*, *Outcome behaviour*, *BCI scenario plan*, *BCI scenario report*, *Behaviour change intervention*, *BCI content*, *BCI dose*, *Behaviour change technique*, *BCI tailoring*, *BCI delivery*, *BCI schedule of delivery*, *BCI mode of delivery*, *BCI style of delivery*, *BCI source*, *BCI engagement*, *BCI context*, *BCI setting*, *BCI social setting*, *BCI physical setting*, *BCI population* and *BCI mechanism of action.* The *BCI comparison evaluation study* has 10 entities: *BCI comparison evaluation study*, *BCI evaluation study*, *BCI study investigator*, *BCI study risk of bias or error*, *BCI evaluation study plan*, *BCI evaluation report*, *BCI study sample*, *BCI evaluation finding*, *BCI outcome estimate* and *BCI effect estimate*. It incorporated planned as well as implemented interventions and methods for evaluating and reporting comparisons.

The coloured circles highlight the plans and reports that relate to BCI scenarios and BCI evaluation studies. As such they have special relationships with these major entities and themselves need to be expanded with planned and reported versions of all the entities in the BCI scenarios and evaluations.

**Table 4.** Relationships used in the BCIO upper level and their mappings to external ontologies.

Relationship	Mapped relationship	ID of mapped relationship	Definition (from mapped source)
1. subclass of	rdfs:subClassOf (equivalent to BFO: is a)	https://www.w3.org/TR/owl-ref/#subClassOf-def	C is_a C _1_ means: for all c, t, if c instance_of C at t then c instance_of C _1_ at t
2. has part	RO: has part	http://purl.obolibrary.org/obo/BFO_0000051	A core relation that holds between a whole and its part
3. has attribute	RO: bearer of	http://purl.obolibrary.org/obo/RO_0000053	A relation between an independent continuant (the bearer) and a specifically dependent continuant (the dependent), in which the dependent specifically depends on the bearer for its existence
4. has process attribute	Maps (with caution, as “process profile” is not unanimously accepted) to BFO: process profile of	http://purl.obolibrary.org/obo/BFO_0000133	b process_profile_of c holds when b proper_occurrent_part_of c& there is some proper_occurrent_part d of c which has no parts in common with b & is mutually dependent on b& is such that b, c and d occupy the same temporal region.
5. has process part	BFO: has occurrent part	http://purl.obolibrary.org/obo/BFO_0000117	Inverse of occurrent_part_of which is defined as: b occurrent_part_of c =Def. b is a part of c & b and c are occurrents.
6. located in	RO: located in	http://purl.obolibrary.org/obo/RO_0001025	A relation between two independent continuants, the target and the location, in which the target is entirely within the location
7. participates in	RO: participates in	http://purl.obolibrary.org/obo/RO_0000056	A relation between a continuant and a process, in which the continuant is somehow involved in the process
8. has study sample	This is a sub-relation of RO:has participant	http://humanbehaviourchange.org/ontology/BCIOR_000001	A relation that holds between a study and the study sample for that study
9. has study investigator	This is a sub-relation of RO:has participant	http://humanbehaviourchange.org/ontology/BCIOR_000002	A relation that holds between a study and the study investigator for that study
10. has BCI source	This is a sub-relation of RO:has participant	http://humanbehaviourchange.org/ontology/BCIOR_000003	A relation that holds between a behaviour change intervention delivery and the source who performs the delivery
11. has BCI context	This is a sub-relation of RO:has participant	http://humanbehaviourchange.org/ontology/BCIOR_000004	A relation that holds between a behaviour change intervention scenario and the context of the scenario
12. has output	RO:has output (which is a sub-relation of RO:has participant)	http://purl.obolibrary.org/obo/RO_0002234	P has output c iff c is a participant in p, c is present at the end of p, and c is not present at the beginning of p.
13. realises	BFO:realizes	http://purl.obolibrary.org/obo/BFO_0000055	To say that b realises c at t is to assert that there is some material entity d & b is a process which has participant d at t & c is a disposition or role of which d is bearer_of at t& the type instantiated by b is correlated with the type instantiated by c. *For example, A BCI Scenario realises a BCI Plan.*
14. realised in	BFO:realized in	http://purl.obolibrary.org/obo/BFO_0000054	Inverse of realises relation.
15. evaluates	This is a sub-relation of IAO:is about	http://humanbehaviourchange.org/ontology/BCIOR_000005	A relation between an evaluation study and the entity being evaluated.
16. comparatively evaluates	This is a sub-relation of IAO:is about	http://humanbehaviourchange.org/ontology/BCIOR_000006	A relation between a comparative evaluation study and the entity being evaluated.
17. difference between	This is a sub-relation of IAO:is about	http://humanbehaviourchange.org/ontology/BCIOR_000007	D is a difference between a and b if d, a and b are data items and d expresses a quantity that differentiates a from b.
18. is about	IAO:is about	http://purl.obolibrary.org/obo/IAO_0000136	Is about is a (currently) primitive relation that relates an information artifact to an entity.
19. has disposition	RO:has disposition	http://purl.obolibrary.org/obo/RO_0000091	A relation between an independent continuant (the bearer) and a disposition, in which the disposition specifically depends on the bearer for its existence

RO: Relation Ontology; rdfs: RDF-Schema; IAO: Information Artifact Ontology; BFO: Basic Formal Ontology.

Each of the entities within the final version of the ontology has a parent class from external ontologies: Basic Formal Ontology (BFO) ( Smith *et al.*, 2005), the top level formal ontology beneath which OBO Foundry ontologies are developed; the Information Artifact Ontology (IAO; Ceusters, 2012), also developed beneath BFO, providing entities of relevance for describing data and information, or the Ontology for Biomedical Investigations (OBI; Bandrowski *et al.*, 2016), with the parent classes being: continuant (BFO), disposition (BFO), generically dependent continuant (BFO), role (BFO), information content entity (IAO), object aggregate (BFO), planned process (OBI) and process (BFO).

### BCIO in context

In addition to discussing the upper level BCIO, the study team discussed the need to represent how entities change over time and the context in which the BCI scenario is embedded. The concept of ‘time’ is represented in several BCIO entities. In the *BCI Scenario*, time is firstly represented in terms of the duration of BCIs and their component BCI sessions or other parts (for example, the time it takes a participant to read a leaflet). Time can also be involved in changes to BCIs as a result of planned adaptations (e.g. the BCI scenario plan entails BCI sources spending more time discussing goals with participants who have difficulties meeting their initial behaviour change targets) or as a result of unplanned changes, e.g. drift in the delivery of the planned length of intervention sessions over time. Outcome behaviours may involve time in terms of their start and end points – for example a person taking a course of medication as prescribed, or in terms of when changes to the rates at which the behaviours are performed occur – such as an intervention leading a person to start going for a walk every day rather than just at weekends.

The BCI schedule involves time in terms of the start and end points when an intervention is first and last implemented (which may be represented by the minute, hour, day, month or year). BCI schedule also encompasses a BCI scenario’s temporal relationship with other BCI scenarios, thus providing a way of capturing complex interdependencies between a given BCI scenario and others that have occurred previously or concurrently. For example, the possibility of a BCI having a greater or smaller impact on the *Outcome behaviour* over the course of the BCI or at different times following the intervention can be captured by specifying the Outcome behaviour follow-up point relative to the start or end of the intervention. Finally, BCI comparison evaluation studies may yield different effect sizes because of study attributes that change over time or are influenced by other studies. For example, a BCI evaluation study may yield different effect sizes because evidence from previous studies has been incorporated in standard treatments.

## Re-use in a separate ontology (AddictO)

To establish that the BCIO upper level was applicable outside of its immediate development context, elements of the ontology were adopted for re-use within AddictO that is being developed separately in parallel with the BCIO. Various elements of BCIO including setting, population and scenario were found to be directly applicable for re-use within AddictO, and have been adopted accordingly. The process of applying the BCIO to re-use in AddictO also helped to clarify the need for parent classes to be defined that generalised beyond behaviour change interventions, for example, *Intervention* as a parent of *Behaviour change intervention*. Including these entities within the upper level BCIO and showing how the BCIO entities fit beneath them helped clarify the definitions of and interrelationships between the BCIO upper level entities in a way that also reduced the problems of circularity in definitions that had been highlighted by expert feedback in an earlier stage of development. It would be good to see the BCIO reused in other application ontologies within the domain to ascertain the extent to which its structure is widely applicable.

## Creation of a sustainability plan

The upper-level BCIO has been made available in the OWL web ontology language and is stored on the HBCP ***GitHub*** repository. It can also be searched and browsed via the Ontology Lookup Service ( Jupp *et al.*, 2015) at https://www.ebi.ac.uk/ols/ontologies/bcio. It is freely available for others to reuse with a CC-BY license version 4.0, in line with the OBO Foundry principle of openness. Once the lower-level ontologies are populated, the full BCIO will be submitted to the OBO Foundry for registration. The GitHub repository includes an ***issue tracker*** portal, allowing feedback with open replies and discussion on the ontology; these can be addressed in subsequent releases of the ontology. GitHub has in-built mechanisms for tracking releases and ***versioning*** as the ontology is revised and updated in response to these discussions and further developments in the field. This will enable the development of tools and interfaces for non-specialists to enable browsing, searching, and viewing the content of the ontologies, both entities and relationships, and associated annotations.

## Discussions and conclusions

The upper level of the BCIO provides an extensive and consistent framework for representing BCIs and their evaluations to help structure thinking and communication about behaviour change interventions. The BCIO forms a composite whole of interrelated lower-level ontologies, with the upper level forming the organising structure that is then populated by entities within each of the lower-level ontologies. The process of developing the lower-level ontologies in turn informs the development of the upper-level ontology, for example, determining gaps where upper-level entities need to be added if it is not possible to classify a lower-level entity appropriately.

The BCIO was developed by a team of behavioural science including a topic-specific (smoking cessation) expert and supported by an ontology expert consultant, as recommended as best practices for the development of ontologies ( Noy & McGuinness, 2001). Recommended practices include structuring according to a standard top-level ontology (BFO), re-use of content and relationships from existing ontologies where possible (such as the Relations Ontology (RO), Information Artifact Ontology (IAO) and Ontology for Biomedical Investigations (OBI)), adopting accepted conventions for naming and defining entities, peer review by external experts, and testing by applying it to annotating evaluation reports.

Although existing ontologies were drawn on where possible, relatively few entities were found relating directly to human behaviour change in existing ontologies. This reflects the fact that the use of ontologies is less widespread in the social and behavioural sciences than in the biological and medical sciences. One challenge faced in defining the entities in BCIO was the need to clarify subtle distinctions between tightly coupled aspects of complex processes, such as between the content of an intervention and its delivery, between dose and scheduling, between intervention population and study sample, and between intervention content and delivery. Expert feedback was very useful. Although some was not relevant to the scope the ontology is supposed to represent, the issues highlighted by the experts will inform future work to provide ontological definitions for core entities in the social and behavioural sciences.

The BCIO incorporates research methods used for evaluation as well as the contexts in which research is conducted and the biases that may result from those. By separating the evaluation study from the BCI scenario, the BCIO explicitly allows for the annotation of attributes of the study and of the study investigator, such as funding sources and competing interests, which may directly or indirectly influence the study outcomes. An entity “BCI study risk of bias or error” is represented as a data item that is about the study and that encapsulates approaches that aim to quantify the likelihood of bias in a study based on a diversity of underlying factors.

As with all ontologies, development is a continuing process and the BCIO upper-level ontology reported here represents a stage in an ongoing activity. Our report of the methods and results chart how we have tackled the challenges; we have also identified further issues to resolve or progress in future. First, expert reviewers noted that the initial version of the ontology focused purely on representation without testing the capabilities of the resulting ontology for automated reasoning to derive inferences based on the represented content. The use of the ontology for more computationally sophisticated purposes is an area that will be addressed in future work. There are several interrelated issues at play, which relate to the fact that the ontology is of course a *representation* of reality, and the adequacy of that representation will be tested in its use. For example, the upper level BCIO will be used as a structure for the annotated HBCP dataset ( Bonin *et al.*, In Press), and the data entities will be mapped against the upper level structure. The aim is to enable researchers and stakeholders to query the data and gain inferences about what might work in particular situations for whom.

Success depends both upon the ontology reflecting the terms and concepts used across primary research and also upon the data entities selected for inclusion in the ontology being those which are responsible for mediating or moderating intervention success. The iterative development of the ontology has been essential to ensure that it corresponds with the way that researchers in the field are carrying out their investigations, so it should reflect their concepts adequately. Knowing whether the categories it contains embody ‘real’ drivers of intervention success and failure is yet to be determined, and it may be possible to assess this only partially, as there are so many possible reasons for apparently similar interventions and contexts to differ from one another that intervention outcomes are affected.

BCI scenarios do not exist in isolation but as part of complex systems. In the current version of the BCIO, each BCI evaluation report is represented as an independent entity describing one or more BCI evaluations. The single trial approach to evaluating BCIs fails to capture possible interactions between BCIs or the evolution of multiple BCIs over time in a complex system. For example, brief opportunistic physician advice on smoking cessation to patients during routine consultations may have a greater impact at a time when there are large increases in tobacco duty and may create a positive feedback cycle leading to greater demand for stop-smoking medicines amplifying the overall impact.

Representing time and context in relation to BCI scenarios is complex. While some aspects of time are represented in the BCIO as noted above, the BCIO as currently formulated includes entities related to BCIs and their study for the purpose of predicting outcome behaviours and effect size estimates. In this approach each BCI scenario and BCI evaluation study is treated as independent. It is desirable to extend this approach to represent changes in entities over time so that one can predict changes in outcomes and effect sizes as a function of continued or repeated application of BCIs, or time since the onset or offset of BCIs, as well as changing context. It is also desirable to be able to predict outcomes and effect sizes from multiple BCIs implemented together or in succession, i.e. forming part of a system.

Nevertheless, the BCIO as presented here contributes to wider developments in representing knowledge in the behavioural sciences. While the scope of the BCIO is limited to the domain of behaviour change, the issues addressed in its development have general relevance for the representation of knowledge about interventions in human populations. It is our hope that this work will lay a foundation for the development of further ontologies of relevance to the social and behavioural research domains in the future.

The BCIO is one of many ongoing efforts to improve reproducibility, organisation and synthesis of evidence in behavioural science and in the biomedical sciences more broadly to enable working across domains and disciplines. For example, the development of the BCIO was informed by the CONSORT guidelines for reporting clinical trials and by the Template for Intervention Description and Replication (TIDieR). By reducing ambiguities and omissions in the reporting and interpreting of BCIs and their evaluations, the BCIO adds value to these reporting guidelines in reducing problems of heterogeneity of reported content and increasing the feasibility of evidence synthesis and scenario prediction, thus making best use of behavioural science knowledge for implementation in policy and practice. 

## Data availability

### Underlying data

**The BCIO is available from:** https://github.com/HumanBehaviourChangeProject/ontologies.

**Archived ontology at time of publication of revised version of the paper:** https://doi.org/10.5281/zenodo.4334592 ( Norris *et al.*, 2020a).

**License:** CC-BY 4.0.

### Extended data

Open Science Framework: Human Behaviour-Change Project. https://doi.org/10.17605/OSF.IO/UXWDB ( West *et al.*, 2020).

This project contains the following extended data related to this method:

- HBCP Ontology Methodology Summary (PDF).
- BCIO Upper Level Expert Feedback (PDF).

Data are available under the terms of the Creative Commons Attribution 4.0 International license (CC-BY 4.0).
