# Supplementary material for: Representation of behaviour change interventions and their evaluation: Development of the Upper Level of the Behaviour Change Intervention Ontology
Source: Wellcome Open Res. 2021 Jan 6;5:123. Originally published 2020 Jun 10. [Version 2] doi: 10.12688/wellcomeopenres.15902.2 (PMC7868854; doi:10.12688/wellcomeopenres.15902.2)
Supplement: Peer review history

# Peer review: Reviewer response for version 2

**DOI:** 10.21956/wellcomeopenres.18213.r41989

**Published:** 2021-02-05

**Authors:** Adrien Barton

**Affiliations:** 1 Institut de recherche en informatique de Toulouse (IRIT), CNRS, Toulouse, France; 2 Interdisciplinary Research Group in Health Informatics (GRIIS), Faculty of Medicine and Health Sciences / Faculty of Sciences, University of Sherbrooke, Quebec, QC, Canada

The authors have revised their paper adequately. The paper is very clear and well organized, and the added examples of entities linked to the Text2Quit interventions greatly help to illustrate the classes of the ontology.

 Here are a few minor remarks:

- The definition of “BCI scenario” reads “A process in which a BCI is applied in a given context […]”. In my first review, I wrote that a BCI, being a process, cannot be “applied”. The authors replied that “the thing that is being applied is the plan”. However, a BCI is not a plan. A possibility would be to change the definition of “BCI scenario” as “A process in which a BCI scenario plan is applied in a given context […]”.
- In the text2Quit example, suppose for simplicity that texts concerning quitting smoking are sent only to two people, Mr. Jones and Mr. Brown. It was not clear for me which of the following processes would then be the instances of *BCI*: sending texts to Mr. Jones; sending texts to Mr. Brown; sending texts to both Mr. Jones and Mr. Brown? (the latter being the mereological sum of the two former processes).
- In the example of *BCI study investigator role*, Abroms and colleagues (mentioned in the Text2Quit example) are not instances of *BCI study investigator role*, but are rather persons who are each bearing such an instance of role.
- I wonder what would be the relation between the *BCI mode of delivery* (which is a process) and the continuants that are used to deliver the BCI. Is the former the history (in BFO’s sense) of the mereological sum of the latter continuants?
- Is there a distinction between “Not smoking for at least 30 days” (instance of *Intervention outcome*) and “Abstaining from smoking for at least […] 30 days” (instance of *Outcome behavior*), as the difference in wording might suggest?
- The article gives an example of *Population behavior* (subclass of *Process*), in the context of Text2Quit, as “The proportion of members of a population who haven’t smoked for at least the last 30 days.” However, it seems difficult to consider a proportion as a process. Do the authors want to refer here to the mereological sum of the processes of abstaining from smoking by some members of the target population?

Is the work clearly and accurately presented and does it cite the current literature?

Yes

If applicable, is the statistical analysis and its interpretation appropriate?

Not applicable

Are all the source data underlying the results available to ensure full reproducibility?

No source data required

Is the study design appropriate and is the work technically sound?

Yes

Are the conclusions drawn adequately supported by the results?

Partly

Are sufficient details of methods and analysis provided to allow replication by others?

Yes

Reviewer Expertise:

Ontology; Ethics of nudges

I confirm that I have read this submission and believe that I have an appropriate level of expertise to confirm that it is of an acceptable scientific standard.

# Peer review: Reviewer response for version 1

**DOI:** 10.21956/wellcomeopenres.17442.r39724

**Published:** 2020-09-02

**Authors:** Adrien Barton

**Affiliations:** 1 Institut de recherche en informatique de Toulouse (IRIT), CNRS, Toulouse, France; 2 Interdisciplinary Research Group in Health Informatics (GRIIS), Faculty of Medicine and Health Sciences / Faculty of Sciences, University of Sherbrooke, Quebec, QC, Canada

This article presents the upper level of BCIO, the Behaviour Change Intervention Ontology, as well as the methodology that has been used to develop it. This article is part of a wider project with parts presented in other papers (such as a preliminary scoping review to identify relevant ontologies and entities). Additional useful documents are also available online (such as a report on experts’ feedback).

 This ontology should be very useful for behavioural and social sciences, and the methodology is sound and very well explained. I have a few reservations concerning definitions of several classes in the ontology, as well as a couple of axioms in the ontology, as explained below.

**Terminology**

 When reading the paper, it was sometimes difficult for me to figure out clearly what would be paradigmatic instances of the classes that are introduced. In particular, several definitions seem to blur the border between processes and Information content entities (ICEs) - see below the detailed comments on specific definitions. Therefore, the authors may consider to provide in their article the analysis of a specific example of BCI, clarifying, in this example, what would be the BCI context, the BCI content, the BCI delivery, the BCI mode of delivery, the BCI schedule of delivery, etc.

 Here are my comments on some of the definitions, in alphabetical order:

BCI context: Is it really true that a BCI context is always independent of the intervention? Isn't it possible for a context to be modified by the intervention?

BCI delivery: "A part of a BCI that is the means by which BCI content is provided."

 I would spontaneously have expected a “means” to be a continuant rather than an occurrent (for example, the mean through which I’m writing this report is a computer). It might be useful to clarify that this is not what is intended by the term “means” in the paper.

BCI dose: "An attribute of BCI content that is its amount or intensity.":

 It may be counter-intuitive that an amount or intensity of a process is itself a process.

 The comment reads: "This is a disjunctive class that is not currently fully defined because specific instances may represent intensity and amount in different ways with different weightings applied to create a metric.” But this seems to mix an amount/intensity with the *representation* of an amount/intensity, which would typically be an ICE.

BCI mode of delivery: "An attribute of a BCI delivery that is the physical or informational medium through which a BCI is provided."

 The term "medium" might suggest that what is defined here is a continuant, but it is actually a process. Here again, some clarification could be useful.

BCI scenario: “A process in which a BCI is applied in a given context, including BCI engagement and outcome behaviour.”

 A BCI is defined by the authors as a process, therefore it cannot be “applied”: it has its own existence that unfolds in time, in a determined spatio-temporal area. What could be “applied” would rather be an ICE that would describe a class of similar BCIs (in which case it is “applied” in the sense of being concretized, in IAO’s vocabulary), or a IAO:Directive Information Entity that would direct one or several BCIs.

BCI scenario plan: "A plan specification that represents an intended or hypothetical BCI scenario."

 Since the authors use a realist framework and define a BCI scenario as a *bona fide* entity, it cannot be "intended or hypothetical": all entities that can be accepted in a realist framework must exist, which excludes “intended entities” or “hypothetical entities” (however, it might perfectly include representation of non-existing entities, as long as those representations exist in someone’s mind or on some representational medium). It rather seems to me that a BCI scenario plan represents a *class* of BCI scenarios, and that such a BCI scenario plan can (but must not) direct one or several BCI scenarios (see the literature on directive informational entities).

BCI schedule: p. 11: "The BCI Schedule: […] Start and end points when an intervention is first and last implemented (the minute, hour, day, month or year)"

 A BCI schedule is defined as a process attribute, which is a process. But a process has no intrinsic connection with "minute, hour, day, month and year", which are representational artifacts created by humans (in a realist framework, a process is independent from how it is represented).

BCI schedule of delivery: “An attribute of a BCI that involves its temporal organisation."

 Here also, spontaneously, I would have imagined a schedule to be an ICE describing the temporal organization of a BCI.

Intervention outcome: “A process that is influenced by an intervention.”

 An intervention can influence many things (and at various levels of granularity) other than the outcome, such as the breathing rhythm of an agent or the trajectory of one of its electrons. To clarify this definition, the authors might therefore add “intentionally”: “A process that is *intentionally* influenced by an intervention.”

Process attribute: “An attribute of a process"

 A few explanations on this notion of “attribute of a process” would be useful (even if we don’t expect in this article a full theoretical treatment of the notion of process attribute, which is highly complex). In particular, what is the difference between a process attribute and a process profile? Some examples of instances of this class would be useful.

**Axioms**

 The ontology available on

https://github.com/HumanBehaviourChangeProject/ontologies/tree/master/Upper%20Level%20BCIO

 features two axioms using the relation “realizes” that seem problematic:

 - ‘BCI evaluation study’ SubClassOf realizes some ‘BCI evaluation study plan’

 - ‘BCI scenario’ SubClassOf realizes some ‘BCI scenario plan’

 ‘BCI evaluation study plan’ and ‘BCI scenario plan’ are subclasses of ‘Plan specification’, which is a subclass of ICE. But the relation realizes is supposed to hold between a process and a realizable entity (see its definition). And an ICE is not a realizable entity.

**Visualization of the taxonomic structure**

 Figure 3 is very useful to visualize the various axioms in the ontology, but does not give a clear overview of the taxonomic structure. An additional schema might be added to describe only the taxonomic structure; or table 3 might be organized in a way that reflects the taxonomic structure, rather than by alphabetical order of the labels.

**Minor comments**

 “upper level ontology” is usually used in the literature as a synonym of “top level ontology” (by contrast to “mid-level ontology” or “domain ontology”), so the formulation “upper level of the Behaviour Change Intervention Ontology (BCIO)” is somewhat idiosyncratic.

 p. 11, section “BCIO in context”: Formatting the text as a list leaves a lot open to interpretation. I would recommend using full sentences to clarify what the author mean exactly here, as some of those points are potentially problematic (cf. my comments above about some potential confusions between processes and ICEs).

 p. 11: "“the all-some rule, so if A has-part B then all instances of A have some instance of B has part” to ensure that the most suitable definitions were selected for the entities.":

 Since the ontology is not written in the OBO language, but in OWL, which admits only relations between particulars (and not between classes), it is not clear to me why introducing the all-some rule here is necessary, or even useful. The only place where I saw it potentially useful is on figure 3, that seems to represent relations between classes. But it might be simpler and clearer to write explicitly, on the legend of the graph, that the arrows r from A to B represents the axiom "A SubClassOf r some B", eschewing relations between classes altogether.

 p. 12, figure 3: I presume that the entity boxes filled with solid colour represent ICEs? This might be added in the legend of the figure.

**References**

 p. 11: "Each of the entities within the final version of the ontology has a parent class from external ontologies: Basic Formal Ontology (BFO) (Smith et al., 2005), […] IAO […] OBI":

 - the Arp, Smith & Spear (2015) reference mentioned elsewhere in the paper might be more appropriate here (and the paper Smith et al. 2005 can be mentioned when introducing the relations).

 - References to ENVO and SEPIO are expected here since some classes have been extracted from those ontologies.

**Typo**

 p. 15: "on ata collections" -> "data"

Is the work clearly and accurately presented and does it cite the current literature?

Yes

If applicable, is the statistical analysis and its interpretation appropriate?

Not applicable

Are all the source data underlying the results available to ensure full reproducibility?

No source data required

Is the study design appropriate and is the work technically sound?

Yes

Are the conclusions drawn adequately supported by the results?

Partly

Are sufficient details of methods and analysis provided to allow replication by others?

Yes

Reviewer Expertise:

Ontology; Ethics of nudges

I confirm that I have read this submission and believe that I have an appropriate level of expertise to confirm that it is of an acceptable scientific standard, however I have significant reservations, as outlined above.

# Response

**Published:** 2020-12-17

**Authors:** Susan Michie

**Affiliations:** University College London, UK

Thank you very much for taking the time to review our paper and for your constructive feedback.  We will address each comment below.

**When reading the paper, it was sometimes difficult for me to figure out clearly what would be paradigmatic instances of the classes that are introduced. In particular, several definitions seem to blur the border between processes and Information content entities (ICEs) - see below the detailed comments on specific definitions. Therefore, the authors may consider to provide in their article the analysis of a specific example of BCI, clarifying, in this example, what would be the BCI context, the BCI content, the BCI delivery, the BCI mode of delivery, the BCI schedule of delivery, etc.**

 Thank you for the suggestion to provide an example of a behaviour change intervention. We now provide an analysis of a specific BCI as part of table 3, providing examples that clarify what would be the BCI context, BCI content and so forth.

**BCI context:**

**Is it really true that a BCI context is always independent of the intervention? Isn't it possible for a context to be modified by the intervention?**

 By “independent” we meant to imply “not dependent,” but realise this could have been clearer.  We have therefore revised the definition to read, “An aggregate of entities that are not dependent on the intervention but may influence the effect of a BCI on its outcome behaviour.”

**BCI delivery: "A part of a BCI that is the means by which BCI content is provided."**

**I would spontaneously have expected a “means” to be a continuant rather than an occurrent (for example, the mean through which I’m writing this report is a computer). It might be useful to clarify that this is not what is intended by the term “means” in the paper.**

 We have replaced the previous definition of BCI delivery with “A part of a BCI that is the process by which BCI content is delivered”

**BCI dose: "An attribute of BCI content that is its amount or intensity.":**

**It may be counter-intuitive that an amount or intensity of a process is itself a process.**

**The comment reads: "This is a disjunctive class that is not currently fully defined because specific instances may represent intensity and amount in different ways with different weightings applied to create a metric.” But this seems to mix an amount/intensity with the *representation* of an amount/intensity, which would typically be an ICE.**

 BCI dose is a process attribute referring to the amount or intensity with which BCI content (specific behaviour change technique (BCT) processes) is delivered.  Variations in amount can involve the number of times particular BCTs are used within a single component of a BCI, for example during one counselling session or in one email, or across the BCI as a whole.  Variations in intensity  can concern the rates at which different BCTs are provided or repeated over the course of a BCI  or, if an intervention used a print mode of delivery, the amount of text and graphics devoted to providing each BCT  (e.g. a leaflet with two sentences on the health benefits of quitting smoking and a leaflet containing 400 words on the health benefits of quitting both implement the BCT “provide information about health consequences”, but the latter does so at a greater intensity)

 We agree that the comment appeared to mix amount/intensity with the representation of an amount/intensity and have therefore revised it to read “This is a disjunctive class that is not currently fully defined because specific BCI content instances may vary in intensity and amount in different ways”

**BCI mode of delivery: "An attribute of a BCI delivery that is the physical or informational medium through which a BCI is provided."  The term "medium" might suggest that what is defined here is a continuant, but it is actually a process. Here again, some clarification could be useful.**

 We have added examples to table to make it clearer what is meant by mode of delivery

**BCI scenario: “A process in which a BCI is applied in a given context, including BCI engagement and outcome behaviour.”**

**A BCI is defined by the authors as a process, therefore it cannot be “applied”: it has its own existence that unfolds in time, in a determined spatio-temporal area. What could be “applied” would rather be an ICE that would describe a class of similar BCIs (in which case it is “applied” in the sense of being concretized, in IAO’s vocabulary), or a IAO:Directive Information Entity that would direct one or several BCIs.**

 The BCI scenario is defined as a planned process, hence the thing that is being applied is the plan. The plan is a realizable entity and while it certainty could be concretized in some sort of “directive information entity” it does not necessarily have to be.

**BCI scenario plan: "A plan specification that represents an intended or hypothetical BCI scenario."**

**Since the authors use a realist framework and define a BCI scenario as a *bona fide* entity, it cannot be "intended or hypothetical": all entities that can be accepted in a realist framework must exist, which excludes “intended entities” or “hypothetical entities” (however, it might perfectly include representation of non-existing entities, as long as those representations exist in someone’s mind or on some representational medium). It rather seems to me that a BCI scenario plan represents a *class* of BCI scenarios, and that such a BCI scenario plan can (but must not) direct one or several BCI scenarios (see the literature on directive informational entities).**

 We have revised the definition of BCI scenario plan to be a subclass of “plan” from Ontology of Biomedical Investigations (OBI).  In OBI, a plan is in the mind of a person, “A plan is a realizable entity that is the inheres in a bearer who is committed to realizing it as a planned process.” 

 The revised definition of “BCI scenario plan” now reads “A plan that is realized in a BCI scenario process”. 

**BCI schedule: p. 11: "The BCI Schedule: […] Start and end points when an intervention is first and last implemented (the minute, hour, day, month or year)"**

**A BCI schedule is defined as a process attribute, which is a process. But a process has no intrinsic connection with "minute, hour, day, month and year", which are representational artifacts created by humans (in a realist framework, a process is independent from how it is represented).**

 We have edited the relevant sentence in the “BCIO in context” section to read, “The BCI schedule involves time in terms of the start and end points when an intervention is first and last implemented (which may be represented by the minute, hour, day, month or year)”

**BCI schedule of delivery: “An attribute of a BCI that involves its temporal organisation."**

**Here also, spontaneously, I would have imagined a schedule to be an ICE describing the temporal organization of a BCI.**

 We agree that there is the potential for the existence of an information content entity, describing the temporal organization of a BCI. However, the temporal organization of a BCI is an entity in its own right, existing regardless of whether it is also codified as an information content entity.  Therefore, we don’t consider “BCI schedule of delivery” to be an information content entity

**Intervention outcome: “A process that is influenced by an intervention.”**

**An intervention can influence many things (and at various levels of granularity) other than the outcome, such as the breathing rhythm of an agent or the trajectory of one of its electrons. To clarify this definition, the authors might therefore add “intentionally”: “A process that is *intentionally* influenced by an intervention.”**

 “Intervention outcome” is proposed to encompass both intended outcomes of an intervention, such as behaviour change or increased quality of life, and unintended intervention outcomes such as treatment side effects or other negative consequences.  Therefore, we do not think adding “intentionally” to the definition reflects our desired meaning.  Instead, we have added to the elaboration, saying “Includes undesirable outcomes, such as treatment side effects, and unintended negative consequences of the intervention.”

**Process attribute: “An attribute of a process"**

**A few explanations on this notion of “attribute of a process” would be useful (even if we don’t expect in this article a full theoretical treatment of the notion of process attribute, which is highly complex). In particular, what is the difference between a process attribute and a process profile? Some examples of instances of this class would be useful.**

 We are using “process attribute” largely synonymously with “process profile”.  As noted, this is a very complex theoretical problem area in ontologies and a full treatment is beyond the scope of this paper.

**Axioms**

**The ontology available on**

**https://github.com/HumanBehaviourChangeProject/ontologies/tree/master/Upper%20Level%20BCIO** **** **features two axioms using the relation “realizes” that seem problematic:**

**- ‘BCI evaluation study’ SubClassOf realizes some ‘BCI evaluation study plan’**

**- ‘BCI scenario’ SubClassOf realizes some ‘BCI scenario plan’**

**‘BCI evaluation study plan’ and ‘BCI scenario plan’ are subclasses of ‘Plan specification’, which is a subclass of ICE. But the relation realizes is supposed to hold between a process and a realizable entity (see its definition). And an ICE is not a realizable entity.**

 We have amended the ontology to define “BCI evaluation study plan” and “BCI scenario plan” as subclasses of “plan” from OBI rather than “plan specification” from IAO.

**Visualization of the taxonomic structure**

**Figure 3 is very useful to visualize the various axioms in the ontology, but does not give a clear overview of the taxonomic structure. An additional schema might be added to describe only the taxonomic structure; or table 3 might be organized in a way that reflects the taxonomic structure, rather than by alphabetical order of the labels.**

 We have re-organised table 3 to better reflect the structure of the ontology

**“Upper level ontology” is usually used in the literature as a synonym of “top level ontology” (by contrast to “mid-level ontology” or “domain ontology”), so the formulation “upper level of the Behaviour Change Intervention Ontology (BCIO)” is somewhat idiosyncratic.**

 We agree that this may be confusing – it is ‘upper’ in relation to the BCIO as a domain-specific ontology. We have added some text to the introduction to make what we mean by the upper level of the BCIO clearer.

**p. 11, section “BCIO in context”: Formatting the text as a list leaves a lot open to interpretation. I would recommend using full sentences to clarify what the author mean exactly here, as some of those points are potentially problematic (cf. my comments above about some potential confusions between processes and ICEs).**

 For the “BCIO in context” section, we have replaced the bullet-pointed list with full sentences.

 p **. 11: "“the all-some rule, so if A has-part B then all instances of A have some instance of B has part” to ensure that the most suitable definitions were selected for the entities.":**

**Since the ontology is not written in the OBO language, but in OWL, which admits only relations between particulars (and not between classes), it is not clear to me why introducing the all-some rule here is necessary, or even useful. The only place where I saw it potentially useful is on figure 3, that seems to represent relations between classes. But it might be simpler and clearer to write explicitly, on the legend of the graph, that the arrows r from A to B represents the axiom "A SubClassOf r some B", eschewing relations between classes altogether**.

 The text cited regarding the “all-some rule” is a direct quote from feedback we received from one of the ontology experts who commented on the BCIO during the development process.  As such we can’t change the wording of this direct quotation, though we agree with the reviewer’s sentiment.

**p. 12, figure 3: I presume that the entity boxes filled with solid colour represent ICEs? This might be added in the legend of the figure.**

 The colouring in of the circles merely constitutes a visual device to highlight the plans and reports that relate to BCI scenarios and BCI evaluation studies – as such they have special relationships with these major entities and themselves need to be expanded with planned and reported versions of all the entities in the BCIO scenarios and evaluations. This has now been added to the legend.

**Typo on p. 15: "on ata collections" -> "data"**

 We have fixed this typo.

# Peer review: Reviewer response for version 1

**DOI:** 10.21956/wellcomeopenres.17442.r39726

**Published:** 2020-08-10

**Authors:** Rebecca C. Jackson

**Affiliations:** 1 Knocean Inc., Toronto, ON, Canada

**Summary:**

- It is difficult to understand what BCIs are effective due to lack of standards in how they are reported. The authors propose the creation of an upper-level ontology (BCIO) to standardize reports of BCIs and integrate the research. The authors introduce the concept of an ontology and do a good job of describing it for an audience who is not knowledgeable in the domain, although the concept of a "logical axiom" may be confusing to some. They proceed to describe BFO, which I assume they use as their top-level. BFO is not universally used as the top-level of an ontology, so I think it would be good for the authors to clarify that.
- The authors very clearly state the goal of BCIO (to answer questions based on integrated evidence). Then, they describe the steps they followed to develop BCIO. They also describe how the ontology was tested to make sure that it makes sense as an application, which is very important for all ontologies, otherwise it is just theoretical work. The testing by annotating published reports proved that it was possible to use BCIO for the intended domain, but I wonder how effective those annotations are for analyzing the data? Perhaps that is for another paper.
- The reuse of BCIO in AddictO also proves that BCIO does a good job of covering the upper-level of the behaviour change domain. It would be good to see BCIO reused in other application ontologies within the domain to really know if it's structure is widely-applicable.
- Finally, the authors talk about how they incorporated changes from the testing and the expert feedback.
- If another group wanted to follow a process for developing an ontology, this paper does an excellent job of outlining the steps for development, testing, and reiteration.

**Comments:**

- There are some very minor grammar issues (e.g. "basic divisions of types of entity in..." should be plural). That said, overall, the writing flows well.
- The BCIO is intended to link "entities in the domain of behaviour change interventions". Typically, upper-level (aka top-level) ontologies are cross-domain, so I'm not sure that I agree with their classification of BCIO as an "upper level" ontology. Their may be some ambiguity in what an "upper level" ontology is between different groups, though. It seems to me that the authors mean that BCIO is a domain ontology. This is just a minor thought, not something that should stop the paper from being indexed.
- I'm not sure if "research article" is quite the correct category for this, since the authors have developed a tool (BCIO). Again, just a comment, not a blocker.
- I answered "Partly" to if this is replicable; other ontology developers would have no need to replicate this exact scenario, but these principles could be applied to other ontologies. Which makes me question further if this is really a "Research" article. That said, I'm not sure what a better category would be.

Is the work clearly and accurately presented and does it cite the current literature?

Yes

If applicable, is the statistical analysis and its interpretation appropriate?

Not applicable

Are all the source data underlying the results available to ensure full reproducibility?

No source data required

Is the study design appropriate and is the work technically sound?

Yes

Are the conclusions drawn adequately supported by the results?

Yes

Are sufficient details of methods and analysis provided to allow replication by others?

Partly

Reviewer Expertise:

Ontology

I confirm that I have read this submission and believe that I have an appropriate level of expertise to confirm that it is of an acceptable scientific standard.

# Response

**Published:** 2020-12-17

**Authors:** Susan Michie

**Affiliations:** University College London, UK

Thank you very much for taking the time to review our paper and for your constructive feedback. We will address each comment below.

**The authors introduce the concept of an ontology and do a good job of describing it for an audience who is not knowledgeable in the domain, although the concept of a "logical axiom" may be confusing to some. They proceed to describe BFO, which I assume they use as their top-level. BFO is not universally used as the top-level of an ontology, so I think it would be good for the authors to clarify that.**

 To reduce potential for confusion, we have replaced “logical axiom” with “logical expression”

 We have also changed the end of the first paragraph in the section “Ontologies” to reflect that BFO is just one top level ontology among others, and that domain ontologies do not have to align with upper level ontologies

**The testing by annotating published reports proved that it was possible to use BCIO for the intended domain, but I wonder how effective those annotations are for analysing the data? Perhaps that is for another paper.**

 We are testing the effectiveness of the prediction system into which the annotation data are fed as part of the Human Behaviour-Change Project evaluation.  We will report the outcomes of this evaluation, once completed, in a separate paper.

**The reuse of BCIO in AddictO also proves that BCIO does a good job of covering the upper-level of the behaviour change domain. It would be good to see BCIO reused in other application ontologies within the domain to really know if its structure is widely-applicable.**

 We agree, and have added a comment to this effect to the paragraph on “re-use in a separate ontology” in the discussion section.

**There are some very minor grammar issues (e.g. "basic divisions of types of entity in..." should be plural). That said, overall, the writing flows well.**

 We have checked for grammar issues and made any required edits throughout the paper.

**The BCIO is intended to link "entities in the domain of behaviour change interventions". Typically, upper-level (aka top-level) ontologies are cross-domain, so I'm not sure that I agree with their classification of BCIO as an "upper level" ontology. There may be some ambiguity in what an "upper level" ontology is between different groups, though. It seems to me that the authors mean that BCIO is a domain ontology.**

 We have now added a sentence to the introduction clarifying what we mean by the upper level of the BCIO, i.e. “the domain-specific entities and their relationships which provide a high-level classification of the components of a behaviour change intervention and serve as a starting point for developing the lower-levels of the  BCIO.”
